# Poly[penta­aqua­tetra­kis(μ_2_-nicotinato-κ^2^ *N*:*O*)(perchlorato-κ*O*)lanthanum(III)disilver(I)]

**DOI:** 10.1107/S1600536809026130

**Published:** 2009-07-11

**Authors:** Biao Guan, Chao-Hua Zhang, Wen-Dong Song

**Affiliations:** a Laboratory and Facility Management Division, Guang Dong Ocean University, Zhanjiang 524088, People’s Republic of China; b School of Food Science and Technology, Guang Dong Ocean University, Zhanjiang 524088, People’s Republic of China; c College of Science, Guang Dong Ocean University, Zhanjiang 524088, People’s Republic of China

## Abstract

In the title complex, [Ag_2_La(C_6_H_4_NO_2_)_4_(ClO_4_)(H_2_O)_5_]_*n*_, the La^III^ atom, lying on a twofold rotation axis, is eight-coordinated by four O atoms from four nicotinate (nic) ligands and four water mol­ecules in a distorted square-anti­prismatic coordination geometry. The Ag^I^ atom is coordinated in an almost linear fashion by two pyridyl N atoms of two nic ligands. The linear coordination is augmented by weak inter­actions with one O atom from a half-occupied ClO_4_^−^ anion and a water mol­ecule lying on a twofold axis. Two Ag(nic)_2_ units connect two La atoms, forming a cyclic unit. These units are further extended into an infinite zigzag chain. The chains are bridged by the disordered perchlorate ions *via* weak Ag—O [2.678 (2) Å] inter­actions. O—H⋯O hydrogen bonds, weak Ag⋯Ag [3.3340 (15) Å] inter­actions and π–π inter­actions between the pyridyl rings [centroid–centroid distance = 3.656 (2) Å] lead to a three-dimensional network.

## Related literature

For related structures see: Evans & Lin (2001 ▶); Luo *et al.* (2004 ▶).
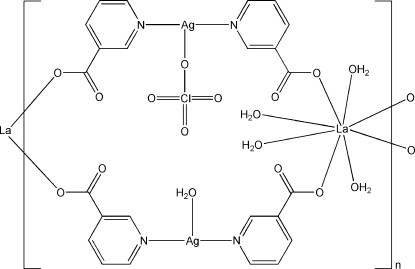


## Experimental

### 

#### Crystal data

[Ag_2_La(C_6_H_4_NO_2_)_4_(ClO_4_)(H_2_O)_5_]
*M* *_r_* = 1032.59
Orthorhombic, 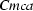

*a* = 35.140 (5) Å
*b* = 12.3371 (16) Å
*c* = 15.046 (2) Å
*V* = 6522.8 (15) Å^3^
*Z* = 8
Mo *K*α radiation
μ = 2.64 mm^−1^
*T* = 298 K
0.30 × 0.25 × 0.22 mm

#### Data collection

Bruker APEXII CCD diffractometer
Absorption correction: multi-scan (*SADABS*; Sheldrick, 1996 ▶) *T*_min_ = 0.465, *T*_max_ = 0.567
15911 measured reflections
2999 independent reflections
2251 reflections with *I* > 2σ(*I*)
*R*_int_ = 0.067

#### Refinement

*R*[*F* ^2^ > 2σ(*F* ^2^)] = 0.049
*wR*(*F* ^2^) = 0.090
*S* = 1.95
2999 reflections
212 parameters
48 restraints
H-atom parameters constrained
Δρ_max_ = 1.90 e Å^−3^
Δρ_min_ = −0.97 e Å^−3^

### 

Data collection: *APEX2* (Bruker, 2007 ▶); cell refinement: *SAINT* (Bruker, 2007 ▶); data reduction: *SAINT*; program(s) used to solve structure: *SHELXS97* (Sheldrick, 2008 ▶); program(s) used to refine structure: *SHELXL97* (Sheldrick, 2008 ▶); molecular graphics: *SHELXTL* (Sheldrick, 2008 ▶) and *DIAMOND* (Brandenburg, 1999 ▶); software used to prepare material for publication: *SHELXTL*.

## Supplementary Material

Crystal structure: contains datablocks I, global. DOI: 10.1107/S1600536809026130/hy2200sup1.cif

Structure factors: contains datablocks I. DOI: 10.1107/S1600536809026130/hy2200Isup2.hkl

Additional supplementary materials:  crystallographic information; 3D view; checkCIF report

## Figures and Tables

**Table 1:** Selected bond lengths (Å)

La1—O1	2.511 (5)
La1—O3^i^	2.401 (4)
La1—O1*W*	2.498 (5)
La1—O2*W*	2.494 (4)
Ag1—N1	2.175 (6)
Ag1—N2	2.161 (6)
Ag1—O6	2.681 (2)
Ag1—O3*W*	2.877 (6)
Ag1—Ag1^ii^	3.3352 (14)

Symmetry codes: (i) 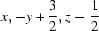; (ii) 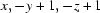.

**Table 2:** Hydrogen-bond geometry (Å, °)

*D*—H⋯*A*	*D*—H	H⋯*A*	*D*⋯*A*	*D*—H⋯*A*
O1*W*—H1*W*⋯O2^iii^	0.86	1.85	2.667 (6)	159
O1*W*—H2*W*⋯O4^ii^	0.84	1.80	2.611 (7)	161
O2*W*—H3*W*⋯O2^iv^	0.84	1.92	2.738 (7)	165
O2*W*—H4*W*⋯O2^v^	0.84	1.89	2.693 (7)	161
O3*W*—H5*W*⋯O1*W*^vi^	0.82	2.11	2.883 (5)	157

Symmetry codes: (ii) 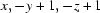; (iii) 
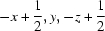
; (iv) 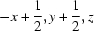; (v) 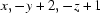; (vi) 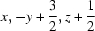.

**Fig. 1.**
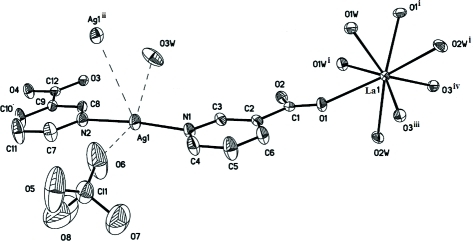
The asymetric unit of the title compound. Displacement ellipsoids are drawn at the 50% probability level. [Symmetry codes: (i) 1/2 - x, y, 1/2 - z; (ii) x, 1 - y, 1 - z; (iii) x, 3/2 - y, -1/2 + z; (iv) 1/2 - x, 3/2 - y, 1 - z.]

**Fig. 2.**
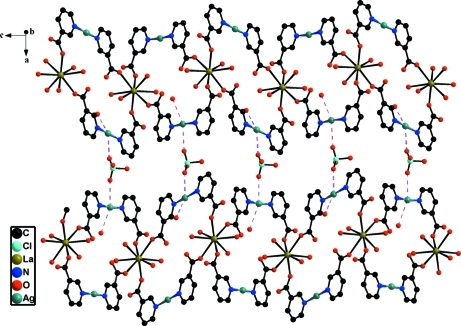
View of the two-dimensional wavelike layer of the title compound. Dashed lines denote weak Ag···O interactions.

**Fig. 3.**
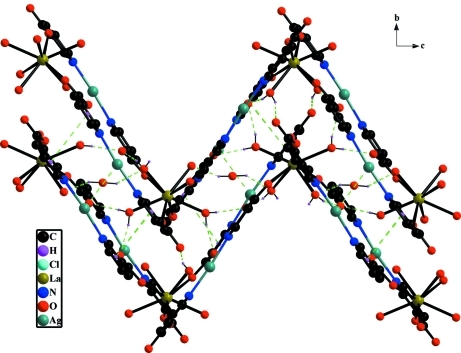
View of the three-dimensional network via hydrogen bonds, weak Ag···O, Ag···Ag, and π–π interactions (dashed lines).

## supplementary crystallographic information

### Comment

In the structural investigation of nictinate complexes, it has been found that nictinate functions as a multidentate ligand with versatile binding and coordination modes (Evans & Lin, 2001; Luo *et al.*, 2004). In this paper, we report the crystal structure of the title compound, a new La^III^ complex, resulted from the hydrothermal treatment of La_2_O_3_, AgNO_3_, perchloric acid and nicotinic acid in water.

As depicted in Fig. 1, the La^III^ atom, lying on a twofold rotation axis, is surrounded by four O atoms from four nic ligands and four water molecules in a distorted square-antiprismatic coordination geometry. The Ag^I^ atom is coordinated in an almost linear fashion by two pyridyl N atoms of two nic ligands. The linear coordination is augmented by weak interactions with one O atom from a half-occupied ClO_4_^-^ anion and a water molecule lying on a twofold rotation axis. The two pyridyl rings of the nic ligands coordinating to the Ag atom are alomost coplanar and have a dihedral angle of 1.74 (2)°. Two Ag(nic)_2_ units connect two La atoms, forming a cyclic unit. These cycles are further extended into an infinite zigzag chain. The chains are bridged by disordered perchlorate ions *via* the weak Ag—O [2.678 (2) Å] interactions into a two-dimensional wavelike layer in the *b* axis direction (Fig. 2). Finally, the layers are further self-assembled into a three-dimensional supramolecular network (Fig. 3) *via* O—H···O hydrogen bonds involving the coordinated water molecules and carboxylate O atoms from the nic ligands (Table 1), weak Ag···Ag [3.3340 (15) Å] interactions and π–π stacking interactions between the pyridyl rings [centroid–centroid distance = 3.656 (2) Å].

### Experimental

A mixture of La_2_O_3_ (0.162 g, 0.5 mmol), AgNO_3_ (0.169 g, 1 mmol), nicotinic acid (0.123 g, 1 mmol), HClO_4_ (0.12 ml) and H_2_O (10 ml) was placed in a 23 ml Teflon-lined reactor, which was heated to 433 K for 3 d, and then cooled to room temperature at a rate of 10 K h^-1^. The pale-purple crystals obtained were washed with water and dried in air (yield 46% based on La).

### Refinement

H atoms on C atoms were positioned geometrically and treated as riding on the parent C atoms, with C—H = 0.93 Å, and with *U*_iso_(H) = 1.2*U*_eq_(C). H atoms of water molecules were located in difference Fourier maps and refined as riding atoms, with *U*_iso_(H) = 1.5*U*_eq_(O). The perchlorate anion is disordered with an occupancy factor of 0.5. The hightest peak in final difference map is located 1.00 Å from La1 and the deepest hole is located 0.94 Å from La1.

### Crystal data

**Table:**

[Ag_2_La(C_6_H_4_NO_2_)_4_(ClO_4_)(H_2_O)_5_]	*F*(000) = 4016
*M**_r_* = 1032.59	*D*_x_ = 2.103 Mg m^−^^3^
Orthorhombic, *C**m**c**a*	Mo *K*α radiation, λ = 0.71073 Å
Hall symbol: -C 2bc 2	Cell parameters from 3600 reflections
*a* = 35.140 (5) Å	θ = 1.4–28°
*b* = 12.3371 (16) Å	µ = 2.64 mm^−^^1^
*c* = 15.046 (2) Å	*T* = 298 K
*V* = 6522.8 (15) Å^3^	Block, colorless
*Z* = 8	0.30 × 0.25 × 0.22 mm

### Data collection

**Table:**

Bruker APEXII CCD diffractometer	2999 independent reflections
Radiation source: fine-focus sealed tube	2251 reflections with *I* > 2σ(*I*)
graphite	*R*_int_ = 0.067
φ and ω scans	θ_max_ = 25.2°, θ_min_ = 2.2°
Absorption correction: multi-scan (*SADABS*; Sheldrick, 1996)	*h* = −42→41
*T*_min_ = 0.465, *T*_max_ = 0.567	*k* = −14→11
15911 measured reflections	*l* = −18→15

### Refinement

**Table:**

Refinement on *F*^2^	Primary atom site location: structure-invariant direct methods
Least-squares matrix: full	Secondary atom site location: difference Fourier map
*R*[*F*^2^ > 2σ(*F*^2^)] = 0.049	Hydrogen site location: inferred from neighbouring sites
*wR*(*F*^2^) = 0.090	H-atom parameters constrained
*S* = 1.95	*w* = 1/[σ^2^(*F*_o_^2^)] where *P* = (*F*_o_^2^ + 2*F*_c_^2^)/3
2999 reflections	(Δ/σ)_max_ = 0.008
212 parameters	Δρ_max_ = 1.90 e Å^−^^3^
48 restraints	Δρ_min_ = −0.96 e Å^−^^3^

### Fractional atomic coordinates and isotropic or equivalent isotropic displacement parameters (Å^2^)

**Table:**

	*x*	*y*	*z*	*U*_iso_*/*U*_eq_	Occ. (<1)
La1	0.2500	1.08273 (5)	0.2500	0.02358 (16)	
Ag1	0.389767 (18)	0.60965 (6)	0.56481 (4)	0.0465 (2)	
C1	0.3039 (2)	0.9196 (6)	0.3991 (4)	0.0299 (18)	
C2	0.34312 (19)	0.8866 (6)	0.4214 (5)	0.0318 (18)	
C3	0.3495 (2)	0.7964 (6)	0.4729 (4)	0.0331 (19)	
H3	0.3287	0.7591	0.4956	0.040*	
C4	0.4138 (2)	0.8140 (8)	0.4627 (5)	0.053 (3)	
H4	0.4380	0.7896	0.4770	0.064*	
C5	0.4097 (2)	0.9060 (8)	0.4114 (7)	0.071 (3)	
H5	0.4311	0.9430	0.3913	0.085*	
C6	0.3744 (2)	0.9422 (7)	0.3905 (5)	0.048 (2)	
H6	0.3714	1.0039	0.3557	0.057*	
C7	0.4322 (2)	0.4349 (7)	0.6693 (6)	0.054 (3)	
H7	0.4534	0.4729	0.6489	0.065*	
C8	0.3685 (2)	0.4135 (6)	0.6768 (5)	0.0313 (18)	
H8	0.3444	0.4376	0.6605	0.038*	
C9	0.37074 (19)	0.3248 (6)	0.7314 (4)	0.0288 (18)	
C10	0.4064 (2)	0.2925 (7)	0.7546 (6)	0.060 (3)	
H10	0.4098	0.2331	0.7919	0.072*	
C11	0.4374 (2)	0.3480 (8)	0.7226 (7)	0.078 (4)	
H11	0.4619	0.3257	0.7375	0.094*	
C12	0.3359 (2)	0.2677 (6)	0.7620 (5)	0.0292 (17)	
N1	0.38410 (17)	0.7606 (5)	0.4913 (4)	0.0369 (16)	
N2	0.39810 (17)	0.4670 (5)	0.6458 (4)	0.0375 (16)	
O1	0.30034 (13)	0.9960 (4)	0.3448 (3)	0.0366 (13)	
O2	0.27688 (13)	0.8707 (4)	0.4351 (3)	0.0331 (13)	
O3	0.30462 (12)	0.3020 (4)	0.7338 (3)	0.0328 (12)	
O4	0.33989 (13)	0.1890 (5)	0.8112 (3)	0.0432 (15)	
O1W	0.28251 (13)	0.9406 (4)	0.1593 (3)	0.0373 (14)	
H1W	0.2679	0.9115	0.1204	0.056*	
H2W	0.2983	0.8959	0.1795	0.056*	
O2W	0.24825 (13)	1.1647 (4)	0.4015 (3)	0.0478 (14)	
H3W	0.2385	1.2269	0.4029	0.072*	
H4W	0.2620	1.1535	0.4461	0.072*	
O3W	0.32305 (14)	0.5000	0.5000	0.074 (3)	
H5W	0.3086	0.5297	0.5356	0.111*	
Cl1	0.5030 (4)	0.6862 (4)	0.5923 (4)	0.0816 (15)	0.50
O5	0.5071 (4)	0.8007 (6)	0.5862 (9)	0.118 (3)	0.50
O6	0.4640 (3)	0.6544 (14)	0.5835 (10)	0.118 (3)	0.50
O7	0.5133 (4)	0.6545 (12)	0.6849 (7)	0.118 (3)	0.50
O8	0.5275 (4)	0.6292 (12)	0.5351 (9)	0.118 (3)	0.50

### Atomic displacement parameters (Å^2^)

**Table:**

	*U*^11^	*U*^22^	*U*^33^	*U*^12^	*U*^13^	*U*^23^
La1	0.0267 (3)	0.0230 (3)	0.0210 (3)	0.000	−0.0014 (3)	0.000
Ag1	0.0542 (4)	0.0386 (4)	0.0466 (4)	0.0050 (4)	−0.0029 (3)	0.0150 (3)
C1	0.043 (4)	0.027 (5)	0.020 (4)	0.006 (4)	−0.007 (4)	−0.007 (3)
C2	0.034 (4)	0.032 (5)	0.028 (4)	−0.004 (4)	−0.004 (4)	−0.004 (4)
C3	0.032 (4)	0.036 (5)	0.030 (4)	0.005 (4)	0.000 (4)	0.010 (4)
C4	0.038 (5)	0.061 (8)	0.060 (6)	−0.003 (5)	−0.004 (4)	0.024 (5)
C5	0.033 (5)	0.075 (9)	0.105 (9)	−0.004 (5)	−0.001 (5)	0.049 (7)
C6	0.044 (5)	0.052 (7)	0.047 (5)	−0.006 (4)	−0.011 (4)	0.029 (4)
C7	0.033 (5)	0.051 (7)	0.078 (7)	−0.004 (4)	−0.002 (5)	0.026 (5)
C8	0.029 (4)	0.037 (5)	0.028 (4)	0.002 (4)	−0.001 (3)	−0.004 (4)
C9	0.033 (4)	0.028 (5)	0.025 (5)	0.003 (4)	−0.006 (3)	−0.001 (4)
C10	0.039 (5)	0.055 (6)	0.087 (7)	−0.002 (4)	−0.007 (5)	0.040 (6)
C11	0.027 (5)	0.069 (8)	0.139 (10)	−0.003 (5)	−0.007 (5)	0.059 (7)
C12	0.035 (4)	0.025 (5)	0.027 (5)	−0.003 (3)	0.004 (4)	−0.010 (4)
N1	0.037 (4)	0.037 (5)	0.037 (4)	0.002 (4)	−0.004 (3)	0.011 (3)
N2	0.040 (4)	0.033 (4)	0.040 (4)	0.004 (3)	0.004 (3)	0.008 (3)
O1	0.041 (3)	0.031 (4)	0.038 (3)	0.006 (2)	−0.005 (3)	0.013 (3)
O2	0.037 (3)	0.036 (4)	0.027 (3)	−0.002 (2)	0.001 (2)	0.005 (2)
O3	0.027 (3)	0.033 (3)	0.038 (3)	0.007 (2)	−0.004 (2)	−0.002 (2)
O4	0.037 (3)	0.038 (4)	0.054 (4)	−0.004 (3)	−0.007 (3)	0.021 (3)
O1W	0.039 (3)	0.034 (4)	0.039 (3)	0.007 (2)	−0.010 (2)	−0.009 (2)
O2W	0.073 (3)	0.046 (4)	0.025 (3)	0.026 (3)	−0.017 (3)	−0.009 (2)
O3W	0.050 (5)	0.123 (10)	0.048 (6)	0.000	0.000	−0.011 (5)
Cl1	0.044 (3)	0.069 (3)	0.132 (4)	0.015 (5)	0.044 (5)	0.014 (3)
O5	0.075 (5)	0.108 (7)	0.172 (8)	0.003 (5)	0.005 (5)	0.035 (6)
O6	0.075 (5)	0.108 (7)	0.172 (8)	0.003 (5)	0.005 (5)	0.035 (6)
O7	0.075 (5)	0.108 (7)	0.172 (8)	0.003 (5)	0.005 (5)	0.035 (6)
O8	0.075 (5)	0.108 (7)	0.172 (8)	0.003 (5)	0.005 (5)	0.035 (6)

### Geometric parameters (Å, °)

**Table:**

La1—O1	2.511 (5)	C7—C11	1.352 (11)
La1—O3^i^	2.401 (4)	C7—H7	0.9300
La1—O1W	2.498 (5)	C8—N2	1.317 (8)
La1—O2W	2.494 (4)	C8—C9	1.371 (10)
Ag1—N1	2.175 (6)	C8—H8	0.9300
Ag1—N2	2.161 (6)	C9—C10	1.360 (9)
Ag1—O6	2.681 (2)	C9—C12	1.484 (10)
Ag1—O3W	2.877 (6)	C10—C11	1.375 (11)
Ag1—Ag1^ii^	3.3352 (14)	C10—H10	0.9300
C1—O1	1.254 (8)	C11—H11	0.9300
C1—O2	1.249 (8)	C12—O4	1.230 (8)
C1—C2	1.475 (9)	C12—O3	1.253 (8)
C2—C6	1.378 (10)	O3—La1^iii^	2.401 (4)
C2—C3	1.373 (10)	O1W—H1W	0.8564
C3—N1	1.324 (8)	O1W—H2W	0.8388
C3—H3	0.9300	O2W—H3W	0.8404
C4—N1	1.306 (9)	O2W—H4W	0.8395
C4—C5	1.379 (11)	O3W—H5W	0.8241
C4—H4	0.9300	Cl1—O8	1.4076
C5—C6	1.354 (10)	Cl1—O5	1.4226
C5—H5	0.9300	Cl1—O6	1.4309
C6—H6	0.9300	Cl1—O7	1.4925
C7—N2	1.312 (9)		
			
O3^iii^—La1—O3^i^	107.4 (2)	N1—C4—H4	119.5
O3^iii^—La1—O2W^iv^	82.65 (16)	C5—C4—H4	119.5
O3^i^—La1—O2W^iv^	69.35 (15)	C4—C5—C6	119.8 (8)
O3^iii^—La1—O2W	69.35 (15)	C4—C5—H5	120.1
O3^i^—La1—O2W	82.65 (16)	C6—C5—H5	120.1
O2W^iv^—La1—O2W	132.1 (2)	C2—C6—C5	119.2 (8)
O3^iii^—La1—O1W	146.78 (15)	C2—C6—H6	120.4
O3^i^—La1—O1W	89.71 (15)	C5—C6—H6	120.4
O2W^iv^—La1—O1W	76.96 (16)	N2—C7—C11	121.5 (8)
O2W—La1—O1W	142.66 (15)	N2—C7—H7	119.2
O3^iii^—La1—O1W^iv^	89.71 (15)	C11—C7—H7	119.2
O3^i^—La1—O1W^iv^	146.78 (15)	N2—C8—C9	124.5 (7)
O2W^iv^—La1—O1W^iv^	142.66 (15)	N2—C8—H8	117.8
O2W—La1—O1W^iv^	76.96 (16)	C9—C8—H8	117.8
O1W—La1—O1W^iv^	90.8 (2)	C10—C9—C8	116.2 (7)
O3^iii^—La1—O1^iv^	75.34 (16)	C10—C9—C12	122.7 (7)
O3^i^—La1—O1^iv^	139.26 (15)	C8—C9—C12	121.1 (6)
O2W^iv^—La1—O1^iv^	70.81 (16)	C9—C10—C11	119.7 (8)
O2W—La1—O1^iv^	132.40 (16)	C9—C10—H10	120.2
O1W—La1—O1^iv^	73.32 (16)	C11—C10—H10	120.2
O1W^iv^—La1—O1^iv^	71.89 (16)	C7—C11—C10	119.7 (8)
O3^iii^—La1—O1	139.26 (15)	C7—C11—H11	120.1
O3^i^—La1—O1	75.34 (16)	C10—C11—H11	120.1
O2W^iv^—La1—O1	132.40 (16)	O4—C12—O3	124.7 (7)
O2W—La1—O1	70.81 (16)	O4—C12—C9	117.9 (7)
O1W—La1—O1	71.89 (16)	O3—C12—C9	117.4 (7)
O1W^iv^—La1—O1	73.32 (16)	C4—N1—C3	119.7 (7)
O1^iv^—La1—O1	129.6 (2)	C4—N1—Ag1	121.8 (5)
N2—Ag1—N1	175.3 (2)	C3—N1—Ag1	118.5 (5)
N2—Ag1—Ag1^ii^	70.66 (16)	C7—N2—C8	118.4 (7)
N1—Ag1—Ag1^ii^	113.42 (17)	C7—N2—Ag1	121.4 (5)
O6—Ag1—N2	88.67 (6)	C8—N2—Ag1	120.0 (5)
O6—Ag1—N1	88.06 (7)	C1—O1—La1	139.5 (5)
O3W—Ag1—N1	98.95 (17)	C12—O3—La1^iii^	150.2 (5)
O3W—Ag1—N2	85.33 (16)	La1—O1W—H1W	113.1
O3W—Ag1—O6	157.70 (7)	La1—O1W—H2W	124.4
O1—C1—O2	124.7 (7)	H1W—O1W—H2W	111.6
O1—C1—C2	116.7 (7)	La1—O2W—H3W	113.8
O2—C1—C2	118.6 (7)	La1—O2W—H4W	130.5
C6—C2—C3	117.6 (7)	H3W—O2W—H4W	111.4
C6—C2—C1	122.1 (7)	O8—Cl1—O5	113.3
C3—C2—C1	120.3 (7)	O8—Cl1—O6	113.1
N1—C3—C2	122.5 (7)	O5—Cl1—O6	111.3
N1—C3—H3	118.7	O8—Cl1—O7	106.8
C2—C3—H3	118.7	O5—Cl1—O7	107.2
N1—C4—C5	121.1 (8)	O6—Cl1—O7	104.5

Symmetry codes: (i) *x*, −*y*+3/2, *z*−1/2; (ii) *x*, −*y*+1, −*z*+1; (iii) −*x*+1/2, −*y*+3/2, −*z*+1; (iv) −*x*+1/2, *y*, −*z*+1/2.

### Hydrogen-bond geometry (Å, °)

**Table:**

*D*—H···*A*	*D*—H	H···*A*	*D*···*A*	*D*—H···*A*
O1W—H1W···O2^iv^	0.86	1.85	2.667 (6)	159
O1W—H2W···O4^ii^	0.84	1.80	2.611 (7)	161
O2W—H3W···O2^v^	0.84	1.92	2.738 (7)	165
O2W—H4W···O2^vi^	0.84	1.89	2.693 (7)	161
O3W—H5W···O1W^vii^	0.82	2.11	2.883 (5)	157

Symmetry codes: (iv) −*x*+1/2, *y*, −*z*+1/2; (ii) *x*, −*y*+1, −*z*+1; (v) −*x*+1/2, *y*+1/2, *z*; (vi) *x*, −*y*+2, −*z*+1; (vii) *x*, −*y*+3/2, *z*+1/2.
